# Invasive Micropapillary Carcinoma of the Breast With Solid Papillary Carcinoma: A Case Report

**DOI:** 10.7759/cureus.39928

**Published:** 2023-06-03

**Authors:** Sohaib Khalid, Aribah Atiq, Fatima Khalid, Faria W Khan, Azra Bashir

**Affiliations:** 1 Histopathology, Chughtai Institute of Pathology, Lahore, PAK

**Keywords:** papillary lesions of breast, rare breast tumors, mixed breast tumors, invasive micropapillary carcinoma, solid papillary carcinoma of

## Abstract

Invasive micropapillary carcinoma and Solid papillary carcinomas are rare histologic subtypes of breast cancer. Co-existence of tumors of the breast like invasive ductal and lobular carcinomas, or invasive ductal carcinoma and mucinous carcinomas have been reported before. But the existence of invasive micropapillary carcinoma with solid papillary carcinoma is a rare occurrence. Here, we are reporting a rare case of a 60-year-old female with a mass in her left breast. The histopathology report showed a tumor containing these two histologic subtypes. Recognition of all tumor subtypes is necessary, as this can impact the treatment strategy.

## Introduction

Invasive micropapillary carcinoma is a rare, distinct, histological subtype of breast carcinoma. Pure micropapillary carcinoma is infrequent, comprising 0.9%-2% of breast carcinomas [1], with the mean age of diagnosis being 50-60 years. According to the literature, in most cases, this tumor type is admixed with invasive breast carcinoma of no special type (NST) or, in the minority of cases, with mucinous carcinoma [2]. Solid papillary carcinoma of the breast is another uncommon malignancy of elderly women, accounting for less than 1% of all breast cancers [3]. Solid papillary carcinoma can exist in both in situ and invasive forms, but, to the best of our knowledge, its co-existence with invasive micropapillary carcinoma has not been reported. This report presents a rare case of invasive micropapillary carcinoma co-existing with solid papillary carcinoma presenting as a lump in the left breast of a 60-year-old woman.

## Case presentation

A 60-year-old woman presented with a history of a lump in her left breast for the last one year. She had no associated symptoms of pain or nipple discharge. There was no history of preceding trauma or infection, but she was initially treated with antibiotics by a local general practitioner. There was no improvement, and she subsequently underwent mammography, which showed a dense irregular mass (breast imaging reporting and data system (BIRADS) V). A wide local excision was performed on her left breast lump, and a biopsy specimen was sent to our center.

**Figure 1:**
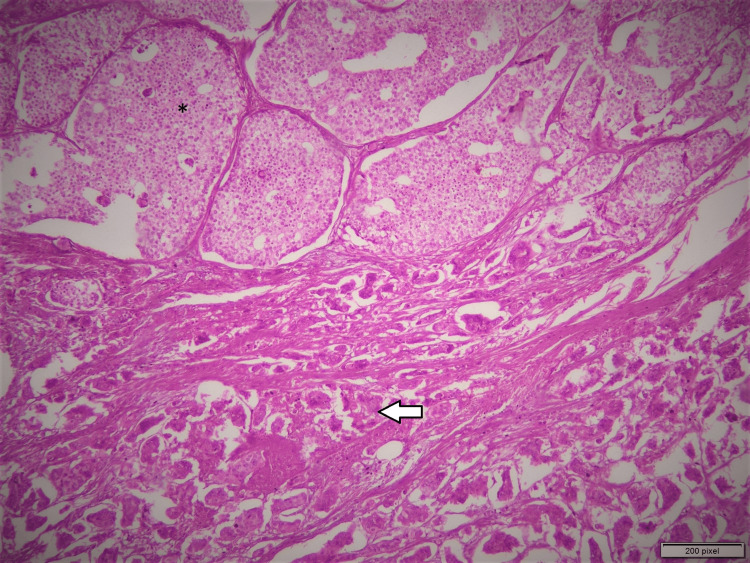
Microscopic photograph of solid papillary carcinoma (asterisk in the upper half of the image). The tumor shows expansile nodules composed of solid epithelial proliferation with delicate fibrovascular cores. The lower half of the image shows invasive micropapillary carcinoma (arrowhead). The tumor is composed of tufts of cells arranged in hollow tubules and morula-like clusters that are surrounded by empty clear spaces.

On macroscopic examination, the specimen was a skin-covered excision measuring 4.0 cm x 3.5 cm x 2.0 cm. Serial slicing revealed a grey-white hard area measuring 1.8 cm x 1.2 cm x 0.8 cm, with a firm tan-white cut surface. Microscopic examination showed two distinct morphological patterns (Figure 1).

One tumor area showed morula-like clusters and hollow tubes surrounded by delicate strands of stroma, as seen in Figures 1 (arrow), 2. Under higher magnification, individual cells showed reverse polarity (i.e., the so-called “inside out” pattern) and abundant eosinophilic cytoplasm. Mitotic figures were also identified. This tumor pattern was diagnosed as invasive micropapillary carcinoma of the breast (grade 2).

**Figure 2:**
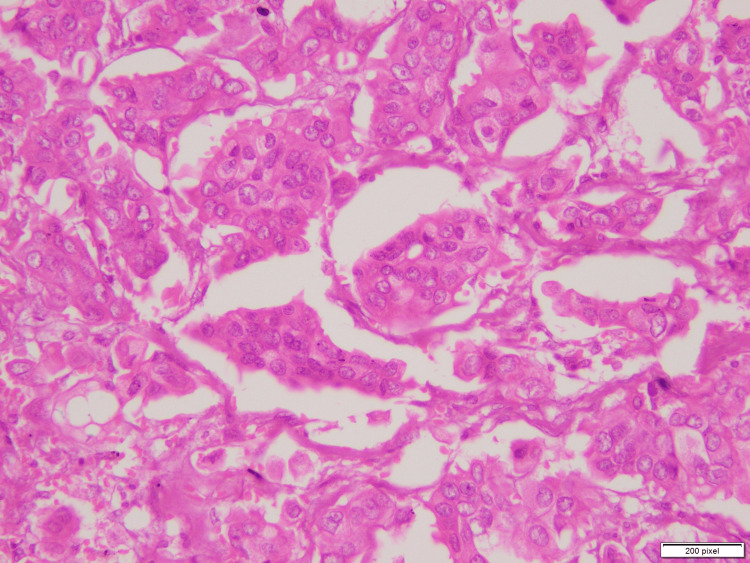
Invasive micropapillary carcinoma showing morula-like clusters surrounded by delicate strands of stroma and individual cells showing reverse polarity.

**Figure 3:**
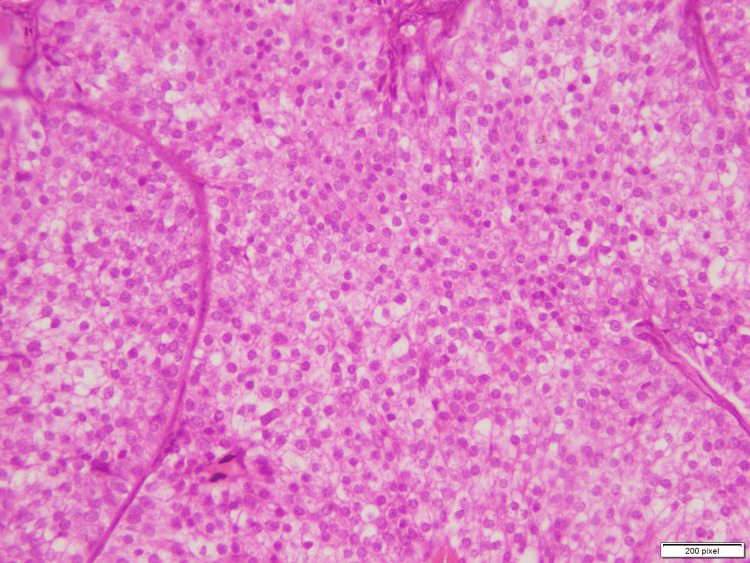
Focus of solid papillary carcinoma showing a monotonous population of round- to spindle-shaped cells arranged in solid nodular architecture with subtle fibrovascular cores.

**Figure 4:**
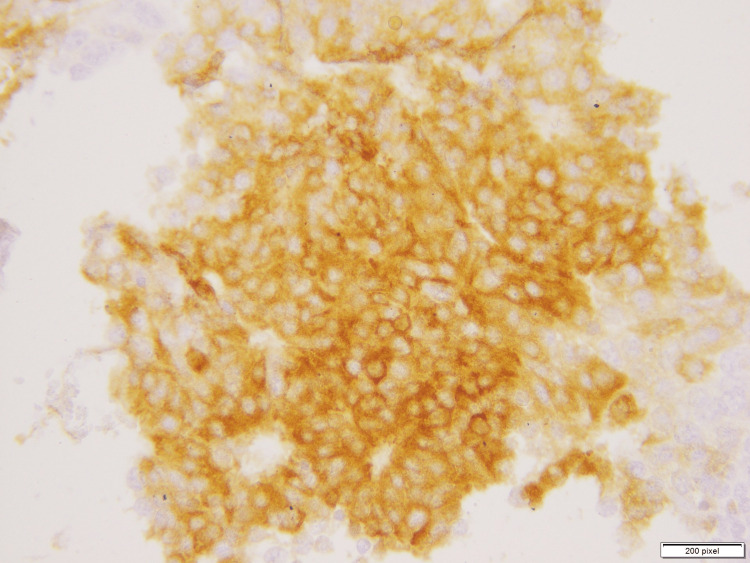
Microscopic photograph of immunohistochemical staining with synaptophysin showing cytoplasmic positivity in the focus of solid papillary carcinoma.

A second tumor area showed expansile nodules composed of a monotonous population of round- to spindle-shaped cells with mild to moderate nuclear atypia and eosinophilic granular cytoplasm, as seen in Figure 3. These cells were arranged in a solid nodular architecture with subtle fibrovascular cores. Individual cells showed neuroendocrine features with dispersed chromatin. Synaptophysin immunostain was applied which diffuse was positive in this tumor focus, as seen in Figure 4. This area was diagnosed as Solid papillary carcinoma.

We reported this case as grade 2 invasive micro papillary carcinoma of the breast with a background 8.0-mm focus of invasive solid papillary carcinoma (grade 2; pathologic stage = pT1c).

## Discussion

Papillary lesions of the breast encompass a wide range of benign and malignant lesions. Invasive micropapillary carcinoma is an aggressive neoplasm associated with an increased incidence of lymphovascular invasion, as compared to invasive carcinoma NST [4]. Therefore, this tumor type is associated with a worse prognosis and a higher rate of metastasis [5]. Solid papillary carcinoma, on the other hand, has a relatively indolent behavior. To the best of our knowledge, these two entities have been reported to co-exist with other tumors, but they have never been reported together.

In a study by Kupik et al., who compared the behavior of pure tumor forms with mixed types, pure invasive micropapillary carcinomas appeared to have more aggressive behavior than that mixed invasive micropapillary carcinoma [6]. Invasive breast carcinoma NST (previously called invasive ductal carcinoma) was found in 86.3% of mixed invasive micropapillary carcinoma cases, followed by invasive lobular carcinoma in 14.7% cases.

Solid papillary carcinomas are another rare subtype of breast carcinoma that can be either in situ or invasive, and they frequently show neuroendocrine differentiation. Commonly, this tumor type co-exists with invasive mucinous carcinomas [7]. A case report by Sharma et al. highlighted the association of solid papillary carcinoma with tubular carcinoma, colloid carcinoma, lobular carcinoma, and the conventional type [8]. As the presence of multiple tumor subtypes can change the treatment strategy, leading to more aggressive therapy, the identification of all patterns is critical.

This unique case report shows a rare association between invasive micropapillary and solid papillary carcinoma. Both histological types showed a distinct morphology in this case, and the diagnosis was confirmed by immunohistochemistry. The patient subsequently underwent a modified radical mastectomy and is currently undergoing chemotherapy.

Although there is a lack of data in the literature about the clinical outcome of mixed breast cancers, their overall behavior is usually associated with the worst type of tumor and the largest tumor size. In our case, the future risk is determined by the invasive micropapillary carcinoma component, which carries a high propensity for lymph node metastasis.

## Conclusions

Solid papillary carcinoma and invasive micropapillary carcinoma are two rare subtypes of papillary lesions of the breast. The co-existence of these two tumors is not a very common phenomenon. The purpose of this case report is to highlight the importance of identifying all breast carcinoma subtypes, as two rare subtypes can co-exist, and prognosis is usually associated with the more aggressive subtype.
